# Prevalence of Low Back Pain and Associated Factors Among Qassim University Medical Students: A Cross-Sectional Study

**DOI:** 10.7759/cureus.44596

**Published:** 2023-09-03

**Authors:** Ahmad H Alwashmi

**Affiliations:** 1 Department of Orthopedic Surgery, College of Medicine, Qassim University, Buraydah, SAU

**Keywords:** saudi arabia, risk factors, prevalence, medical students, low back pain

## Abstract

Introduction

Low back pain (LBP) is a widespread and incapacitating issue that impacts a considerable portion of the adult population. Medical students, physicians, and other healthcare professionals have a high incidence of LBP. This study aimed to determine the prevalence and associated factors of LBP among medical students at two medical colleges in the Kingdom of Saudi Arabia.

Methods

Participants in this online cross-sectional study were medical students in two medical colleges at Qassim University in the Kingdom of Saudi Arabia. A questionnaire and the Oswestry Disability Index were sent through a social media platform. SPSS was used to analyze the data with a significance level of p < 0.05.

Results

The data of 350 medical school students were evaluated. Most participants were males (n = 180, 51.4%), 21 to 23 years old (n = 190, 54.3%), first-year medical students (n = 108, 30.9%), and in the basic medical education phase (n = 228, 65.3%). LBP prevalence was 82%. We found that 72.6% (n = 254) of participants did not exercise or participate in outdoor sports. More than half of the participants reported using a computer or laptop for fewer than eight hours per day. LBP was significantly associated with BMI (F = 3.457, p = 0.017) and computer use duration (T = 3.695, p < 0.001). LBP was not significantly associated with age (F = 0.892, p = 0.411) or gender (T = 1.566, p = 0.118). More than 90% (n = 323) of students had no disability per the Oswestry Disability Index.

Conclusion

LBP was highly prevalent among medical students and more prevalent among females, though gender and LBP were not associated. LBP was associated with high BMI and prolonged computer usage. Stakeholders should work to raise students’ awareness of LBP and methods to improve their lifestyles and behaviors.

## Introduction

Low back pain (LBP) is defined as a nagging, dull ache in the back from below the lower margins of the twelfth ribs to the gluteal folds [1]. LBP is often followed by discomfort in one or both legs and can sometimes result in neurological symptoms in the legs. LBP can be due to a variety of causes, including poor posture and stress on muscles in the region [2].

LBP is a common health issue among working-age people, and its prevalence rises with age. A study reported that the worldwide point prevalence of activity-limiting LBP was 7.3%, making LBP among the leading causes of disability worldwide [3]. According to a 2019 survey, the prevalence of LBP ranges from 1.4% to 20% in Canada, the USA, and Europe [4]. A systematic review found that the prevalence of LBP ranged from 64% to 89% across various professions in a working-age population in the Kingdom of Saudi Arabia [5].

LBP is often dependent on the nature of a person’s job or profession. It is, thus, highly prevalent among healthcare professionals due to the requirements of their professions. A systematic review of 154 studies revealed an estimated lifetime prevalence of LBP in healthcare personnel of 54.8% [6]. LBP prevalence has been found to be higher among dentists and nurses than among other health professionals, and ophthalmologists specializing in surgery have been shown to experience LBP more frequently (53%) than professionals who perform other medical duties [7,8].

In addition to doctors and healthcare professionals, medical students experience high rates of LBP, with causes that include smoking, stress, poor sleeping posture, and family history [9]. One study showed a 75.8% LBP prevalence among Belgrade medical students, with higher percentages among female medical students due to mental stress, continuous sitting, fatigue, and a lack of exercise [10]. Another study showed that almost 72% of medical students suffered from LBP due to stress and numerous hours of study and training [11].

Despite the documentation of LBP prevalence in numerous populations in the literature, no prior studies have been carried out in the Qassim region of Saudi Arabia; thus, the pattern and prevalence of LBP in medical students in this region are not fully known. The current investigation aimed to evaluate the prevalence of LBP among medical students in two medical institutions in the Kingdom of Saudi Arabia and identify the factors related to LBP prevalence. Ultimately, the study aims to enhance future health workers’ awareness of modifiable risk factors.

## Materials and methods

Study design

This was a cross-sectional study.

Study sample

All medical students from Unaizah and Buriydah medical colleges at Qassim University were included. Students were contacted via a social media platform (WhatsApp) and asked to participate by completing the online Oswestry Disability Index and a questionnaire relating to their demographic characteristics. A reminder was sent fortnightly to all students.

Survey instrument

In the current study, we adopted the previously validated Oswestry Disability Index [11]. Participants were additionally asked about their demographics, health behaviors, lifestyle, pain intensity score, and the characteristics and effects of their LBP. Several modifications were made to the demographic questionnaire for the purposes of this study, including the addition of a question asking if participants had ever sought medical advice for LBP.

Sample-size calculation

The estimated sample size for a precision of 5% and a prevalence of 48% in a population of 2,000 was 322. This calculation was based on a 95% confidence interval (CI) with limits of 43%-53%. The CI provides a range of values likely to contain the true value of the population with a certain degree of confidence. In this case, the CI was 95%.

Data collection

Online surveying was conducted using a Google Forms survey. All participants were informed of the study’s goals before their consent was obtained. Data were collected over one month from June 1, 2022, to June 30, 2022. Data were kept confidential and were only made public for research purposes.

Statistical analysis

Online data were gathered, cleaned, and imported to a Microsoft Excel spreadsheet before being analyzed using IBM SPSS Statistics for Windows, version 22.0 (IBM Corp., Armonk, USA). The findings of the descriptive analysis were presented as frequencies and percentages for categorical variables and as means and standard deviations for continuous variables. Analysis of variance and t-tests were performed to determine associations. Relevant statistics and p-values were used to present the results of these tests. The significance level used for all tests was p < 0.05.

## Results

Data from 389 medical college students were collected. After exclusions due to incomplete responses, data from 350 students were analyzed. The majority of participants (n = 180, 51.4%) were males between the ages of 21 and 23 years, first-year medical students (n = 108, 30.9%), and in the basic phase of medical education (n = 228, 65.1%) (Table 1).

 

**Table 1:** Demographic Characteristics of the Study Participants

Variable	Frequency	Percentage
Gender		
Male	180	51.4
Females	170	48.6
Age Group		
18-21	125	35.7
21-23	190	54.3
24 and above	35	10.0
Academic Year		
1st year	108	30.9
2nd year	67	19.1
3rd year	53	15.1
4th year	68	19.4
5th year	54	15.4
Phase		
Basic	228	65.1
Clinical	122	34.9
BMI		
Underweight	13	3.7
Normal	242	69.1
Overweight	50	14.3
Obese	45	12.9

Participants showed wide variability in habits and lifestyles. We observed that 72.6% (n = 254) of the medical students did not participate in exercise or sports, with only 27.4% (n = 96) participating in exercise and sports. Of the 96 participants engaging in exercise, 34 (35.4%) reported spending one-two hours, 38 (39.6%) reported spending three-four hours, and 24 (25%) reported spending more than five hours participating in sports or exercise weekly. We found that 5.1% (n = 18) of the medical students were smokers, 4.3% (n = 15) were former smokers, and 90.6% (n = 317) were non-smokers. We also observed that 54.6% (n = 191) of participants spent less than eight hours per day using a computer or laptop.

When using a laptop, most participants preferred the sitting position (n = 277, 79%), followed by the recumbent position (n = 61, 17.4%); only 12 (3.4%) participants preferred other positions, including lying on the side, sitting on the floor, and resting a leg on the table. In sleeping habits, 53.1% (n = 186) of participants reported sleeping four to six hours per day and 30.9% (n = 108) of participants reported sleeping seven to eight hours per day. Back discomfort while in bed was reported by 160 (45.7%) participants (Table 2).

**Table 2:** Habits and Lifestyle of the Study Participants

Habits and Lifestyle	Frequency	Percentage
Do you practice any exercises/sports currently?		
No	254	72.6
Yes	96	27.4
Duration of exercise/sports (hours in a week)		
1-2	34	35.4
3-4	38	39.6
5-7	24	25.0
Smoker		
Yes	18	5.1
No	317	90.6
Ex-smoker	15	4.3
How many hours do you usually spend using computers or tablets daily?		
Up to 8 hours	191	54.6
More than 8 hours	159	45.4
In which position do you use your computers or tablets?		
Other	12	3.4
Sitting position	277	79.1
Recumbent position (lying down on your back or abdomen)	61	17.4
How many hours do you sleep per night?		
< 4 hours	19	5.4
4-6 hours	186	53.1
7-8 hours	108	30.9
8 hours +	37	10.6
Do you feel any back discomfort while in bed?		
Yes	160	45.7
No	190	54.3

**Figure 1:**
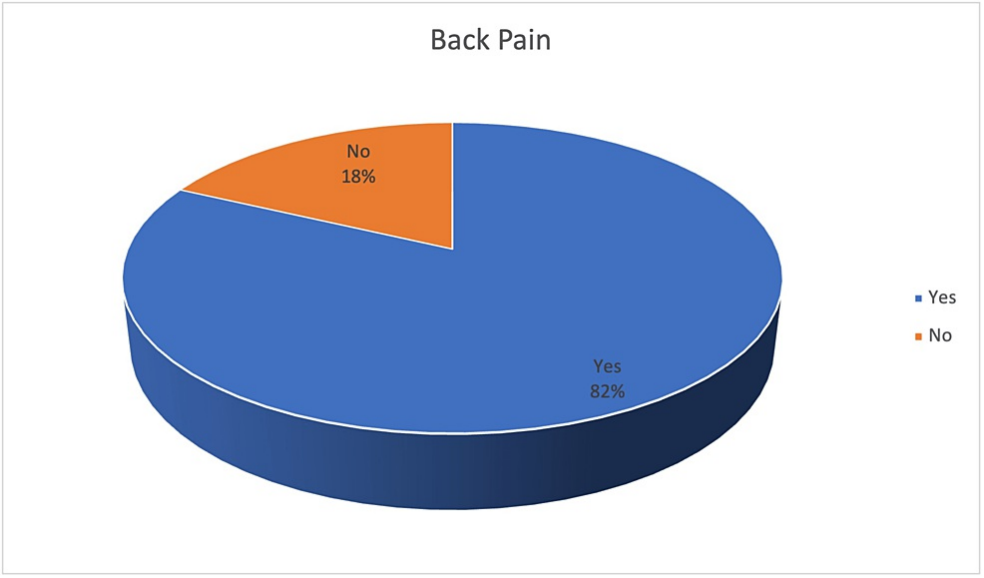
Prevalence of LBP among the Study Participants LBP: Low back pain

LBP was reported by 287 (82%) participants, while 63 (18%) participants stated that they had never felt LBP (Figure 1).

Of the 287 participants who reported LBP, 49.5% (n = 142) further stated that their pain duration was less than three months, while 32.1% (n = 92) reported suffering from LBP for more than a year. Of the participants with LBP, 56.1% (n = 161) had pain for one to seven days, and 20.6% (n = 59) suffered for more than 30 days. Despite the pain, only 10.5% of the study participants visited a doctor, physical therapist, or chiropractor for treatment. Out of 287 participants with LBP, 48.1% (n = 138) reported no pain currently. Work and leisure activities were impacted by LBP in 44.9% (n = 129) and 39.9% (n = 114) of participants, respectively (Table 3).

**Table 3:** Characteristics and Effects of LBP LBP: Low back pain

Characteristics and Effects of LBP	Frequency N (Denominator)	Percentage
For how long you have had back pain?		
Less than 3 months	142	49.5
Less than 6 months	20	7.0
Less than 1 year	33	11.5
More than 1 year	92	32.1
Total	287	100.0
What is the total length of time that you had LBP during the last 12 months?		
0 days	14	4.9
1-7 days	161	56.1
8-30 days	53	18.5
>30 days	59	20.6
Have you been to any doctor, physiotherapist, chiropractor, or related person for LBP during the last 12 months?		
Yes	30	10.5
No	257	89.5
Pain intensity now?		
I have no pain now	138	48.1
Very mild	107	37.3
Moderate	36	12.5
Fairly severe	4	1.4
Very severe	2	0.7
Has LBP caused you to reduce your work activity (at home or in college) during the last 12 months?		
Yes	129	44.9
No	158	55.1
Has LBP caused you to reduce your leisure activity?		
Yes	114	39.9
No	172	60.1

**Figure 2:**
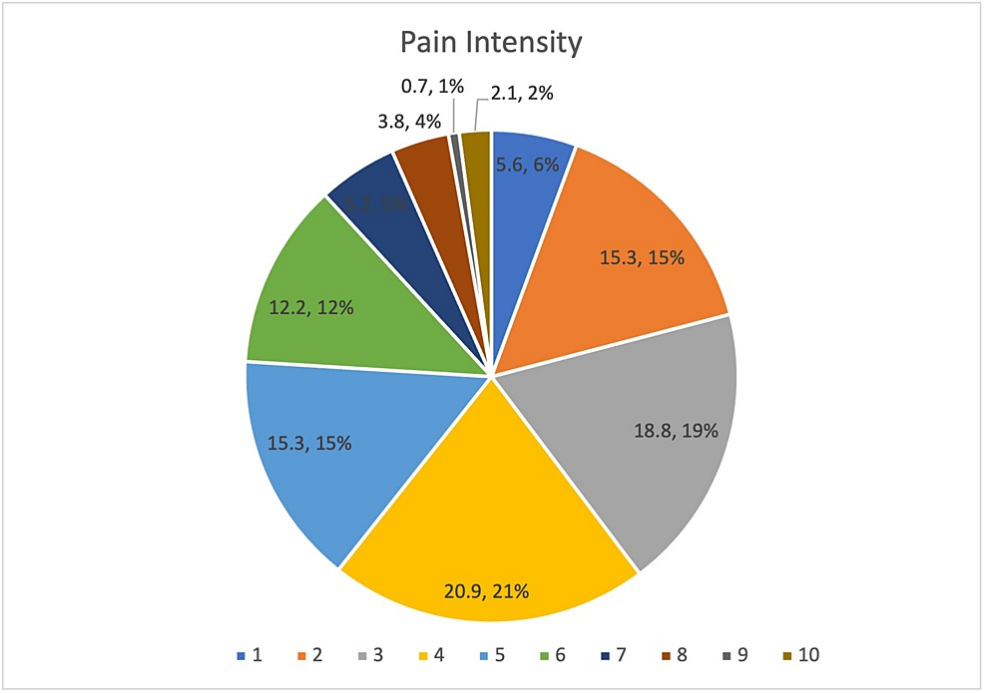
Pain Intensity Score (0-10) of the Study Participants

When characterizing pain severity, 283 participants reported pain intensity levels greater than 0, of which 218 (76%) participants categorized their pain intensity as between the levels of 1 and 5. A pain intensity of 4 was the most commonly reported pain level (n = 60, 20.9%). Six (2.1%) participants reported a pain severity of 10 out of 10 (Figure 2).

The relationship between LBP and various demographic and lifestyle factors was investigated. LBP was not significantly associated with age (F = 0.892, p = 0.411). However, 78.4% (n = 98) of participants who were 18 to 20 years old reported LBP, while 85.7% (n = 30) of participants who were 24 years or older reported LBP. Females had a higher prevalence of LBP compared to males, though the difference was not significant (T = 1.566, p = 0.118). No significant difference was observed between students in the basic and clinical phases of their medical education (T = 0.197, p = 0.844) (Table 4).

**Table 4:** Association of LBP with Demographics and Lifestyle LBP: Low back pain

LBP	Total	Yes	NO	F or T, P Value
Age				
18-20	125	98 (78.4%)	27 (21.6%)	F = 0.892, P = 0.411
21-23	190	159 (83.7%)	31 (16.3%)	
24 and above	35	30 (85.7%)	5 (14.3%)	
Gender				
Male	180	142 (78.9%)	38 (21.1%)	T = 1.566 , P = 0.118
Females	170	145 (85.3%)	25 (14.7%)	
BMI				
Underweight	16	13 (81.3%)	3 (18.8%)	F = 3.457, P = 0.017
Normal	239	186 (77.8%)	53 (22.2%)	
Overweight	47	43 (91.5%)	4 (8.5%)	
Obese	48	45 (93.8%)	3 (6.3%)	
Phase				
Basic	224	183 (81.6%)	41 (18.3%)	T = 0.197, P = 0.844
Clinical	126	104 (82.5%)	22 (17.4%)	
Exercise				
Yes	96	87 (90.6%)	9 (9.4%)	T = -3.013, P = 0.003
No	254	200 (78.7%)	54 (21.3%)	
Computer usage				
Up to 8 hours per day	191	144 (75.4%)	47 (24.6%)	T = 3.695, P < 0.001
More than 8 hours per day	159	143 (89.9%)	16 (10.1%)	
In which position do you use your computers or tablets?				
Sitting position	277	225 (81.2%)	52 (18.8%)	F = 1.372, P = 0.255
Recumbent position lying down on your back or abdomen)	61	50 (82.0%)	11 (18%)	
Other	12	12 (100%)	0 (0%)	

BMI was significantly associated with LBP (F = 3.457, p = 0.017). While 77.8% (n = 186) of individuals with normal BMI had LBP, 81.3% (n = 13) of students who were underweight, 91.5% (n = 43) of students who were overweight, and 93.8% (n = 45) of students who were obese reported LBP (Table 4).

LBP was significantly more prevalent among those who reported exercising or being involved in sports (T = -3.013, p = 0.003). Of the 350 medical students, only 96 (27.42%) were involved in exercise or sports; of these students, 87 (90.6%) reported LBP. On the other hand, LBP was reported by 78.7% (n = 200) of students who did not engage in sports activities (Table 4).

A significant correlation was found between LBP and the duration of computer usage (T = 3.695, p < 0.001). Pain was more prevalent among those who reported using a computer or laptop for more than 8 hours a day. Body position during computer usage was not associated with LBP (F = 1.372, p = 0.255) (Table 4).

**Figure 3:**
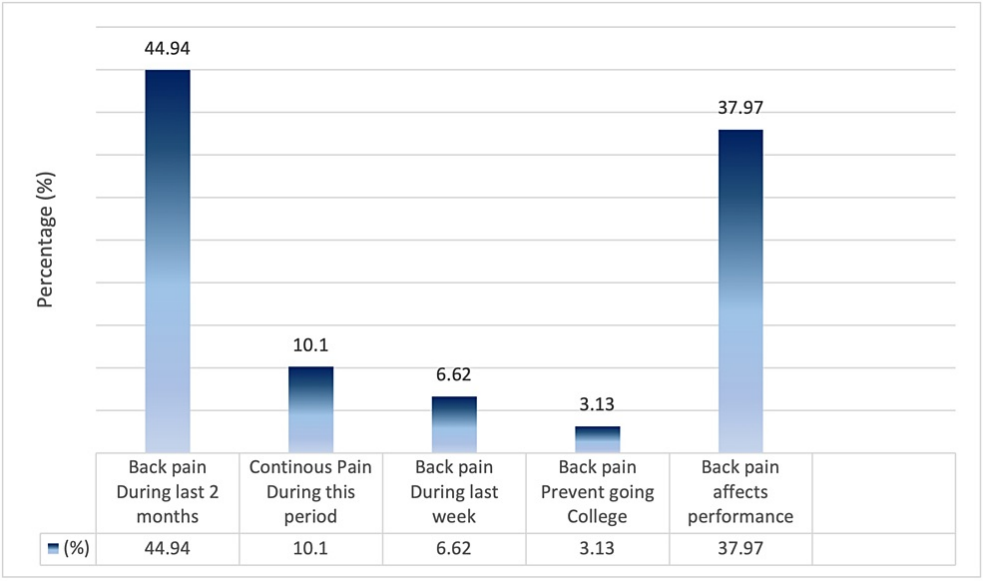
Back Pain History among the Medical Students

When asked about the prevalence and impact of LBP, 44.94% (n = 129) of participants reported experiencing LBP in the preceding two months, with 10.10% (n = 29) indicating that LBP was a persistent issue. Moreover, 6.62% (n = 19) reported an onset of pain within the past week. Of those with a history of LBP, 3.13% (n = 9) required absences from work due to pain. Furthermore, 37.97% (n = 109) noted that pain impacted their academic performance (Figure 3).

According to the Oswestry Disability Index, 92.23% (n = 323) of students had no disability, 6.57% (n = 23) of students had mild disability, and 1.14% (n = 04) of students had moderate disability. No participants reported severe or complete disability.

## Discussion

The current study aimed to determine the prevalence of LBP among students in two medical colleges at Qassim University. In addition, the associations between LBP and factors including demographics, BMI, habits, and lifestyle were studied.

In this study, LBP was prevalent among medical college students, and female students were more affected. Interest in sports and physical activity among the students was low. In addition, LBP was associated with a high BMI and extended computer use. A direct correlation was observed between exercise and LBP.

According to the findings of this study, 82.0% of medical students reported having LBP at some point in their lives. These findings are consistent with previous studies conducted in different parts of the world. A study of 629 Brazilian medical students revealed that 81.7% of students reported having LBP [12]. A Serbian study found that 75.8% of medical students had LBP at some point in their lives [10]. A Pakistani study reported that over the course of a year, 67.28% of medical students experienced pain in their lower back [13]. A recent study performed among medical students of Umm Al-Qura University, Makkah, Saudi Arabia, found that 75.7% of participants reported LBP [14]. However, a study in Taif reported a lower percentage of 33.3% of medical students with LBP [15]. There are numerous potential reasons for these disparities in LBP prevalence, including differences in race, heredity, lifestyle, study duration, sitting positions, and sleeping patterns. 

LBP was prevalent among females in the current study. Previous research supports these findings [10,12,13]. Some researchers have attributed this tendency to women’s lifestyle choices including poor posture and prolonged sitting [16,17]. In the current study, no significant differences were noted among different age groups. A similar finding was observed in a study conducted on 1,800 medical students in the USA that assessed the prevalence of LBP in the same age groups and found no association between age and LBP [18].

Our study revealed that almost half of the medical students surveyed experienced LBP within the past months. Our findings support previous research demonstrating that LBP was reported by 56.6% of medical students [19]. However, the prevalence of LBP among teachers in another study was reported to be 67.3%, a substantially higher value than that found in our study, which may be due to differences in the age groups surveyed [20].

Nearly half of the participants reported a duration of LBP of less than three months, while one-third reported LBP for over a year. Similar findings were reported in a recent study, where most medical students had LBP for less than three months [14]. In the current study, more than half of the respondents reported that LBP persisted for one to seven days, and 27% stated that LBP remained for more than 30 days. Over half of the subjects reported not currently experiencing LBP. Overall, these findings are consistent with the results reported in previous studies on medical students [10,14,15,19].

Our results revealed that more than two-thirds of medical students did not engage in sports or other forms of physical activity. Although long-term studies suggest that sedentary behavior may have consequences for LBP rather than being a risk factor, cross-sectional studies indicate a significant association between sedentary behavior and LBP. There is weak evidence that previous sedentary activity increases the risk of developing future LBP [21,22]. Unlike the findings of several previous studies [10,22], our study found that medical students who exercised had a significantly higher prevalence of LBP. A study including undergraduate students from a sports and physical education institute found a strong association between certain sports and LBP [23]. As poor posture and exercise technique may contribute to LBP, this might explain the association between LBP and exercise in our study sample.

Similar to a previous survey’s finding that 41.4% of medical students used computers for more than 10 hours per day, 45.4% of medical students in the current study reported using computers or laptops for more than eight hours per day [19]. We found a significant association between long hours of computer use and LBP, which is in line with previous studies associating prolonged use of a computer or laptop with LBP and linking extensive computer use to a variety of musculoskeletal disorders [24]. Students who spend more than eight hours on a computer or tablet have been reported to have a significantly higher frequency of LBP than other groups [25]. Previous studies have additionally found that prolonged periods of sitting exacerbate spinal compression, which is relevant to the findings of the current study, as most students preferred a sitting position followed by a recumbent position while using a computer [25,26]. However, no significant differences were seen based on position in the current study, though LBP was prevalent in individuals who chose other positions while using a computer, including a recumbent position or horizontal position on the side, back, or abdomen. Improper sitting behavior has also been linked to chronic LBP [27].

The current study found that 69% of medical students had a normal BMI, while 12.3% were obese. Another Saudi Arabian study on medical students revealed similar rates of normal and obese individuals [14]. We found a positive association between BMI and LBP, with obese and overweight individuals showing a significantly higher prevalence of LBP. These results are consistent with previous study findings of increased LBP incidence in people who are overweight and obese [28]. A meta-analysis of ten cohort studies with 29,748 participants was conducted comparing people of normal weight with those who were overweight or obese. It was found that being overweight or obese can elevate the likelihood of developing LBP. The pooled odds ratios for overweight and obesity were 1.15 (95% CI, 1.08-1.21) and 1.36 (95% CI, 1.18-1.57), respectively [29]. Moreover, being overweight or obese has been correlated with seeking treatment for LBP and having persistent LBP [28].

Based on the results of the Oswestry Disability Index, approximately 99% of medical students demonstrated no or mild levels of disability. These findings are consistent with a previously conducted study on medical students, which revealed a low proportion of individuals exhibiting moderate disability and none reporting severe or complete disability [30].

Strengths and limitations

This was a multi-center study that included medical students of different levels. While this study relied on anonymous self-reports, it is challenging to rule out the possibility of information bias. The current study may be affected by sampling bias, as only interested students completed the online survey form. Furthermore, participants could have overestimated or underestimated their LBP.

Although self-reported surveys can be erroneous, medical students are able to comprehend the definitions employed in the study and the need to provide accurate and honest responses to health surveys due to their medical knowledge. However, the findings may not be representative of all Saudi medical college students. Finally, the cross-sectional methodology of our investigation has the established drawbacks associated with this study design in finding factors related to LBP, including raised BMI, prolonged computer usage, and improper sitting positions.

## Conclusions

In conclusion, this survey found that more than 80% of medical students complained of LBP, which was more prevalent among females and primarily associated with high BMI, prolonged computer usage. A lack of exercise in conjunction with long study hours was frequently mentioned. However, the high prevalence of reported LBP notwithstanding, there was no significant impact on academic performance. Stakeholders should work to raise students’ awareness of LBP and methods to improve lifestyles and behaviors associated with LBP.

## References

[1] Hartvigsen J, Natvig B, Ferreira M (2013). Is it all about a pain in the back?. Best Pract Res Clin Rheumatol.

[2] Hartvigsen J, Hancock MJ, Kongsted A (2018). What low back pain is and why we need to pay attention. Lancet.

[3] GBD 2015 Disease and Injury Incidence and Prevalence Collaborators (2016). Global, regional, and national incidence, prevalence, and years lived with disability for 310 diseases and injuries, 1990-2015: a systematic analysis for the Global Burden of Disease Study 2015. Lancet.

[4] Fatoye F, Gebrye T, Odeyemi I (2019). Real-world incidence and prevalence of low back pain using routinely collected data. Rheumatol Int.

[5] Aldera MA, Alexander CM, McGregor AH (2020). Prevalence and incidence of low back pain in the Kingdom of Saudi Arabia: a systematic review. J Epidemiol Glob Health.

[6] Rezaei B, Mousavi E, Heshmati B, Asadi S (2021). Low back pain and its related risk factors in health care providers at hospitals: a systematic review. Ann Med Surg (Lond).

[7] Çınar-Medeni Ö, Elbasan B, Duzgun I (2017). Low back pain prevalence in healthcare professionals and identification of factors affecting low back pain. J Back Musculoskelet Rehabil.

[8] Bertelmann T, Heutelbeck A, Bopp S (2021). Prevalence of back pain among German ophthalmologists. Ophthalmic Res.

[9] Ilic I, Milicic V, Grujicic S, Zivanovic Macuzic I, Kocic S, Ilic MD (2021). Prevalence and correlates of low back pain among undergraduate medical students in Serbia, a cross-sectional study. PeerJ.

[10] Vujcic I, Stojilovic N, Dubljanin E, Ladjevic N, Ladjevic I, Sipetic-Grujicic S (2018). Low back pain among medical students in Belgrade (Serbia): a cross-sectional study. Pain Res Manag.

[11] Davidson M, Keating J (2005). Oswestry disability questionnaire (ODQ). Aust J Physiother.

[12] Tavares C, Salvi CS, Nisihara R, Skare T (2019). Low back pain in Brazilian medical students: a cross-sectional study in 629 individuals. Clin Rheumatol.

[13] Pal S, Ali K, Khaqan MB, Gul H, Javed S (2022). Prevalence of low back pain in medical students of United Medical and Dental College Karachi. J Pak Orthop Assoc.

[14] Goweda RA, Idris KJ, Bakhsh AJ (2020). Prevalence and associated risk factor of low back pain among medical student of Umm Al-Qura University, Makkah, Saudi Arabia: cross-sectional study. Med Sci.

[15] Alturkistani LH, Hendi OM, Bajaber AS, Alhamoud MA, Althobaiti SS, Alharthi TA, Atallah AA (2020). Prevalence of lower back pain and its relation to stress among medical students in Taif University, Saudi Arabia. Int J Prev Med.

[16] Ozcan Kahraman B, Kahraman T, Kalemci O, Salik Sengul Y (2018). Gender differences in postural control in people with nonspecific chronic low back pain. Gait Posture.

[17] Bento TP, Genebra CV, Maciel NM, Cornelio GP, Simeão SF, Vitta A (2020). Low back pain and some associated factors: is there any difference between genders?. Braz J Phys Ther.

[18] Amelot A, Mathon B, Haddad R, Renault MC, Duguet A, Steichen O (2019). Low back pain among medical students: a burden and an impact to consider!. Spine (Phila Pa 1976).

[19] AlShayhan FA, Saadeddin M (2018). Prevalence of low back pain among health sciences students. Eur J Orthop Surg Traumatol.

[20] Al-Rowayeh MA, Al-Sabt YA, Moustafa MA, Al-Qareer AH, Al-Anzi MM, Moussa MA (2017). Low back pain among high school teachers in Kuwait: prevalence, risk factors and level of disability. KMJ.

[21] Chen SM, Liu MF, Cook J, Bass S, Lo SK (2009). Sedentary lifestyle as a risk factor for low back pain: a systematic review. Int Arch Occup Environ Health.

[22] Citko A, Górski S, Marcinowicz L, Górska A (2018). Sedentary lifestyle and nonspecific low back pain in medical personnel in north-east Poland. Biomed Res Int.

[23] Triki M, Koubaa A, Masmoudi L, Fellmann N, Tabka Z (2015). Prevalence and risk factors of low back pain among undergraduate students of a sports and physical education institute in Tunisia. Libyan J Med.

[24] Kanchanomai S, Janwantanakul P, Pensri P, Jiamjarasrangsi W (2012). Prevalence of and factors associated with musculoskeletal symptoms in the spine attributed to computer use in undergraduate students. Work.

[25] Todd AI, Bennett AI, Christie CJ (2007). Physical implications of prolonged sitting in a confined posture-a literature review. Ergonomics SA: J Ergonomics Soc S Afr.

[26] Park JH, Srinivasan D (2021). The effects of prolonged sitting, standing, and an alternating sit-stand pattern on trunk mechanical stiffness, trunk muscle activation and low back discomfort. Ergonomics.

[27] Bontrup C, Taylor WR, Fliesser M, Visscher R, Green T, Wippert PM, Zemp R (2019). Low back pain and its relationship with sitting behaviour among sedentary office workers. Appl Ergon.

[28] Shiri R, Karppinen J, Leino-Arjas P, Solovieva S, Viikari-Juntura E (2010). The association between obesity and low back pain: a meta-analysis. Am J Epidemiol.

[29] Zhang TT, Liu Z, Liu YL, Zhao JJ, Liu DW, Tian QB (2018). Obesity as a risk factor for low back pain: a meta-analysis. Clin Spine Surg.

[30] Boszczowski N, Pinto RCR, de Araújo Junior FA (2021). Low back pain in medical students: prevalence and related factors. Coluna/Columna.

